# Staging for Breast Cancer With Internal Mammary Lymph Nodes Metastasis: Utility of Incorporating Biologic Factors

**DOI:** 10.3389/fonc.2020.584009

**Published:** 2021-01-14

**Authors:** Chen-Lu Lian, Hai-Yan Zhang, Jun Wang, Jian Lei, Li Hua, Yong-Xiong Chen, San-Gang Wu

**Affiliations:** ^1^Department of Radiation Oncology, The First Affiliated Hospital of Xiamen University, Xiamen, China; ^2^The Sixth People’s Hospital of Huizhou, Affiliated Huiyang Hospital of Southern Medical University, Huizhou, China; ^3^Department of Obstetrics and Gynecology, The First Affiliated Hospital of Xiamen University, Xiamen, China; ^4^Eye Institute of Xiamen University, Fujian Provincial Key Laboratory of Ophthalmology and Visual Science, School of Medicine, Xiamen University, Xiamen, China

**Keywords:** breast cancer, internal mammary lymph nodes, staging, prognosis, American Joint Committee on Cancer

## Abstract

**Purpose:**

To validate the 8th edition of the American Joint Committee on Cancer (AJCC) pathological prognostic staging system for breast cancer patients with internal mammary lymph nodes (IMN) metastasis (N3b disease, stage IIIC in 7th AJCC anatomical staging).

**Methods:**

Breast cancer patients with IMN metastasis diagnosed between 2010 and 2014 were retrieved from the Surveillance, Epidemiology, and End Results program. Chi-squared test, Log-rank test, Kaplan-Meier method, and Cox proportional hazard analysis were applied to statistical analysis.

**Results:**

We included 678 patients with N3b disease in this study. Overall, 68.4% of patients were downstaged to IIIA and IIIB diseases from the 7th anatomical staging to 8th pathological prognostic staging. The new pathological prognostic staging system had better discriminatory value on prognosis prediction among IMN-metastasized breast cancer patients, with a 5-year breast cancer-specific survival (BCSS) of 92.7, 77.4, and 66.0% in stage IIIA, IIIB, and IIIC diseases, respectively (P<0.0001), and the 5-year overall survival (OS) rates was 85.9, 72.1, and 58.7%, respectively (P<0.0001). The results of the multivariate prognostic analysis showed that the new pathological prognostic staging was the independent prognosis related to BCSS and OS, the 8th AJCC pathological prognostic stages showed worse BCSS and OS with gradually increased hazard ratios.

**Conclusion:**

The 8th AJCC pathological prognostic staging system offers more refined prognostic stratification to IMN-metastasized breast cancer patients and endorses its use in routine clinical practice for this specific* *subgroup* *of* *breast* *cancer.

## Introduction

Breast cancer (BC) is most frequently diagnosed in females and the leading cause of female cancer death in the majority of countries around the world (1). BC is prone to have lymphatic metastasis at early stages due to its abundant lymphatic drainage, which mainly includes the axillary lymph node (ALN) area and internal mammary lymph node (IMN) area. The majority of breast lymph drainage runs into the ALN chain, of which 9–45% also flows into the IMN chain (2). A literature review, including 9,817 BC patients, showed that 2.9–32.7% of patients presented IMN metastasis (3). It was estimated that 28–52% of patients with ALN metastasis might also have IMN metastasis, while the incidence of IMN metastasis decreased to 5–17% in patients with ALN-negative disease (4). There were significantly different disease characteristics between patients with and without IMN metastasis (5). IMN metastasis was more likely to occur in BC patients with advanced disease, medially located tumors, and ALN metastasis (6, 7). However, the detection and treatment of IMN metastasis have long been debated, and the impact of IMN metastasis on the prognostic assessment in BC patients remains controversial. Previous studies have demonstrated that IMN-metastasized BC has a worse prognosis, whereas several studies showed that IMN metastasis was not correlated with an inferior outcome (5, 8–10). Therefore, biological heterogeneity* *may be presented for this population, and the traditional primary tumor (T)-regional lymph nodes (N)-distant metastasis (M) staging system could no longer accurately predict the survival outcomes of IMN-metastasized BC patients in the era of biomarkers.

In the traditional anatomical staging, IMN metastasis was staged as IIIC regardless of the T category. Although multiple revisions have been made to reflect the improvements in diagnosis and treatment since the AJCC breast cancer staging system was first published, the biologic factors were not integrated into the staging system until the 8th American Joint Committee on Cancer (AJCC) BC staging manual was introduced (11). In the 8th AJCC BC staging manual, the important biologic factors in BC, including grade, estrogen receptor (ER) status, progesterone receptor (PR) status, and human epidermal growth factor receptor 2 (HER2) status, were integrated into this newly revised staging system (12). For BC patients who received standardized systemic therapy, this staging system could provide better stratification regarding prognostic prediction (11), and several validated studies have confirmed the better performance in the prognostic assessment of the new staging compared to the 7th staging (13–21). However, studies regarding the validation of the new staging system for IMN-metastasized breast cancer are limited. A recent study from Korea analyzed the value of prognostic prediction of the newly proposed 8th AJCC pathological prognostic staging system in IMN-metastasized BC, but only 66 patients were included (16). Therefore, this retrospective study aimed to evaluate the accuracy of the 8th AJCC pathological prognostic stages in the prognostic assessment of the IMN-metastasized BC patients using the data from the Surveillance, Epidemiology, and End Results (SEER) program.

## Materials and Methods

### Patients

Women diagnosed with BC from 2010 to 2014 were identified using the SEER database. SEER program was established by United States (US) National Cancer Institute as a cancer registry, which collected cancer data on demographics, clinicopathological features, the first course of therapy, and follow-up for vital status (22). We identified patients who met the following criteria: T1-4N3bM0 BC with IMN metastasis; treatment with breast-conservation surgery or mastectomy; available variables including age, race/ethnicity, marital status, histology, histologic grade, T category, ER status, PR status, HER2 status, adjuvant radiotherapy, and chemotherapy. The definition of the N3b included metastases to the axillary lymph nodes and in the presence of positive ipsilateral internal mammary lymph node metastases by sentinel node biopsy, fine needle aspiration/core needle biopsy, or imaging. Patients aged <18 years, non-positive pathological diagnoses, and unavailable for local treatment procedures were excluded. There is no need to apply for approval from the Institutional Review Board of the First Affiliated Hospital of Xiamen University due to the de-identified information in the SEER database.

### Variables

The following variables were included: age, race/ethnicity, marital status, histology, histologic grade, T category, ER status, PR status, HER2 status, surgery methods, the receipt of chemotherapy, and radiotherapy. The pathological prognostic stages were assigned according to the newly proposed AJCC BC pathological prognostic staging manual, and the T category was according to the 7th AJCC staging system (12).

### Statistical Analysis

Chi-square test or fisher’s exact test was used to compare patients’ characteristics after stratification by stage change. The area under the curve (AUC) was used to investigate the discriminatory ability of the 8th AJCC pathological prognostic stages in predicting survival outcomes using the receiver operating characteristics (ROC). Survival curves were drawn using the Kaplan–Meier method, and the significant differences among different stages were compared using the log-rank test. Multivariate Cox regression analysis was used to determine the independent prognostic factors associated with breast cancer-specific survival (BCSS) and overall survival (OS). All statistical analyses were conducted by the IBM SPSS 26.0 software package (IBM Corp., Armonk, NY). P values <0.05 were considered statistically significant.

## Results

A total of 678 breast cancer patients were included. The median age of the cohort was 52 years (range, 20–93 years). Four hundred and seventeen (61.5%) patients were white, and 366 (54.0%) patients were married. T categories were T1 in 106 (15.7%), T2 in 266 (39.2%) patients, T3 in 154 (22.7%) patients, and T4 in 152 (22.4%) patients. ER, PR, and HER2 status was positive in 431 (63.5%), 322 (47.5%), and 191 (28.2%) patients, respectively. A total of 622 (91.7%) patients received chemotherapy, and 485 (71.5%) patients received adjuvant radiotherapy. Overall, 30 (4.4%), 203 (30.0%), and 445 (65.6%) patients were well-differentiated (G1), moderately differentiated (G2), and poorly/undifferentiated (G3) tumors, respectively. The detailed patient and tumor characteristics are shown in **Table 1**.

**Table 1:** Patient characteristics.

Variables	N (%)	N			P
		IIIA	IIIB	IIIC	
Age (years)					0.326
<50	286(42.2)	69	118	99	
≥50	392(57.8)	98	179	115	
Race/ethnicity					
Non-Hispanic White	417 (61.5)	117	189	111	0.001
Non-Hispanic Black	109 (16.1)	14	44	51	
Hispanic (All Races)	85 (12.5)	23	31	31	
Other	67 (9.9)	13	33	21	
Histology					<0.001
IDC	544 (80.2)	107	243	194	
ILC	41 (6.1)	23	14	4	
Other	93 (13.7)	37	40	16	
Histologic grade					<0.001
G1	30 (4.4)	23	7	0	
G2	203 (30.0)	144	48	11	
G3	445 (65.6)	0	242	203	
T category					0.086
T1	106 (15.7)	30	45	31	
T2	266 (39.2)	77	106	83	
T3	154 (22.7)	36	73	45	
T4	152 (22.4)	24	73	55	
Breast surgery					0.335
BCS	136 (20.1)	36	52	48	
MAST	542 (79.9)	131	245	166	
Adjuvant RT					0.192
No	193 (28.5)	51	91	51	
Yes	485 (71.5)	116	206	163	
Chemotherapy					<0.001
No	56 (8.3)	21	30	5	
Yes	622 (91.7)	146	267	209	
HER2 status					<0.001
Negative	487 (71.8)	148	125	214	
Positive	191 (28.2)	19	172	0	
ER status					<0.001
Negative	247 (36.4)	0	91	156	
Positive	431 (63.6)	167	206	58	
PR status					<0.001
Negative	356 (52.5)	0	146	210	
Positive	322 (47.5)	167	151	4	

ILC, infiltrating lobular carcinoma; IDC, infiltrating ductal carcinoma; BCS, breast-conserving surgery; ER, estrogen receptor; G1, well-differentiated; G2, moderately differentiated; G3, poorly/undifferentiated; HER2, human epidermal growth factor receptor-2; MAST, mastectomy; RT, radiotherapy; PR, progesterone receptor; T, tumor.

According to the traditional anatomical stages, N3b patients were staged as the IIIC stage, regardless of the T category. Among the 678 patients in the anatomic IIIC stage, 464 patients (68.4%) had their stage reassigned and all of them were downstaged, whereas 214 patients (31.6%) remained unchanged according to the novel AJCC staging system. These patients were separated into three stages of IIIA (n=167, 24.6%), IIIB (n=297, 43.8%), and IIIC (n=214, 31.6%) according to the 8th edition criteria. All patients in G1 disease and 94.6% of patients in G2 disease were downstaged, whereas 45.6% of those in G3 disease were unchanged. In addition, all patients with double hormone receptors (HoR)-positive disease (ER-positive and PR-positive) were downstaged, whereas 49.6% of the single* *HoR-positive disease (ER-positive or PR-positive) patients were unchanged. Furthermore, all patients with HER2 positivity were downstaged, 45.0% of which were double HoR-negative disease (ER-negative and PR-negative). Regarding the breast cancer subtype, 99.3% of the triple-negative (TNBC) patients had their stage unchanged, and only 1 TNBC patient was downstaged. Although 70.8, 68.8, 70.8, and 63.8% of patients in T1, T2, T3, and T4 categories were downstaged, respectively, there was no statistical significance in the distribution among the stage migration. Tumor characteristics of stage migration are presented in **Table 2**.

**Table 2:** Comparisons of tumor characteristics among stage change.

Variables	Down stage (%)	No change (%)	Up stage (%)	P
Histologic grade				<0.001
G1	30 (100)	0 (0)	0	
G2	192 (94.6)	11 (5.4)	0	
G3	242 (54.4)	203 (45.6)	0	
HoR status				<0.001
ER positive and PR positive	314 (100)	0 (0)	0	
ER positive and PR negative	59(50.4)	58(49.6)	0	
ER negative and PR positive	4 (50.0)	4 (50.0)		
ER negative and PR negative	87 (36.4)	152 (63.6)	0	
HER2 status				<0.001
HER2 positive	191 (100)	0 (0)	0	
HER2 positive, ER negative, and PR negative	86 (100)	0 (0)	0	
HER2 positive, ER positive, and PR negative	36(100)	0(0)	0	
HER2 positive, ER negative, and PR positive	4(100)	0(0)	0	
HER2 positive, ER positive, and PR positive	65 (100)	0 (0)	0	
HER2 negative	273 (56.1)	214 (43.9)	0	
HER2 negative, ER negative, and PR negative	1 (0.7)	152 (99.3)	0	
HER2 negative, ER positive, and PR negative	23 (28.4)	58 (71.6)	0	
HER2 negative, ER negative, and PR positive	0 (0)	4 (100)	0	
HER2 negative, ER positive, and PR positive	249 (100)	0 (0)	0	
T category				0.538
T1	75 (70.8)	31 (29.2)	0	
T2	183 (68.8)	83 (31.2)	0	
T3	109 (70.8)	45 (29.2)	0	
T4	97 (63.8)	55 (36.2)	0	

ER, estrogen receptor; G1, well-differentiated; G2, moderately differentiated; G3, poorly/undifferentiated; HER2, human epidermal growth factor receptor-2; HoR, hormone receptors; PR, progesterone receptor; T, tumor.

**Figure 1:**
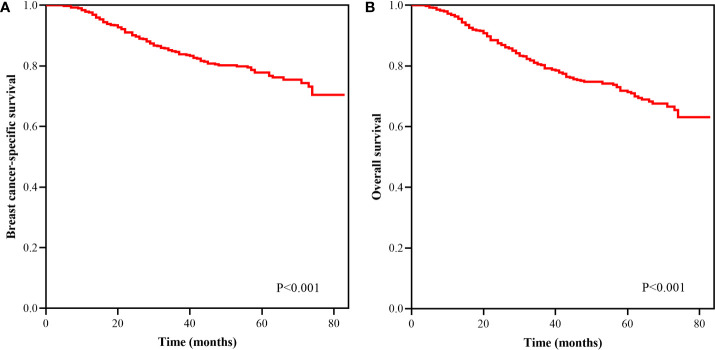
Kaplan–Meier survival curves of breast cancer-specific survival **(A)** and overall survival **(B)** in breast cancer patients with internal mammary lymph nodes metastasis.

**Figure 2:**
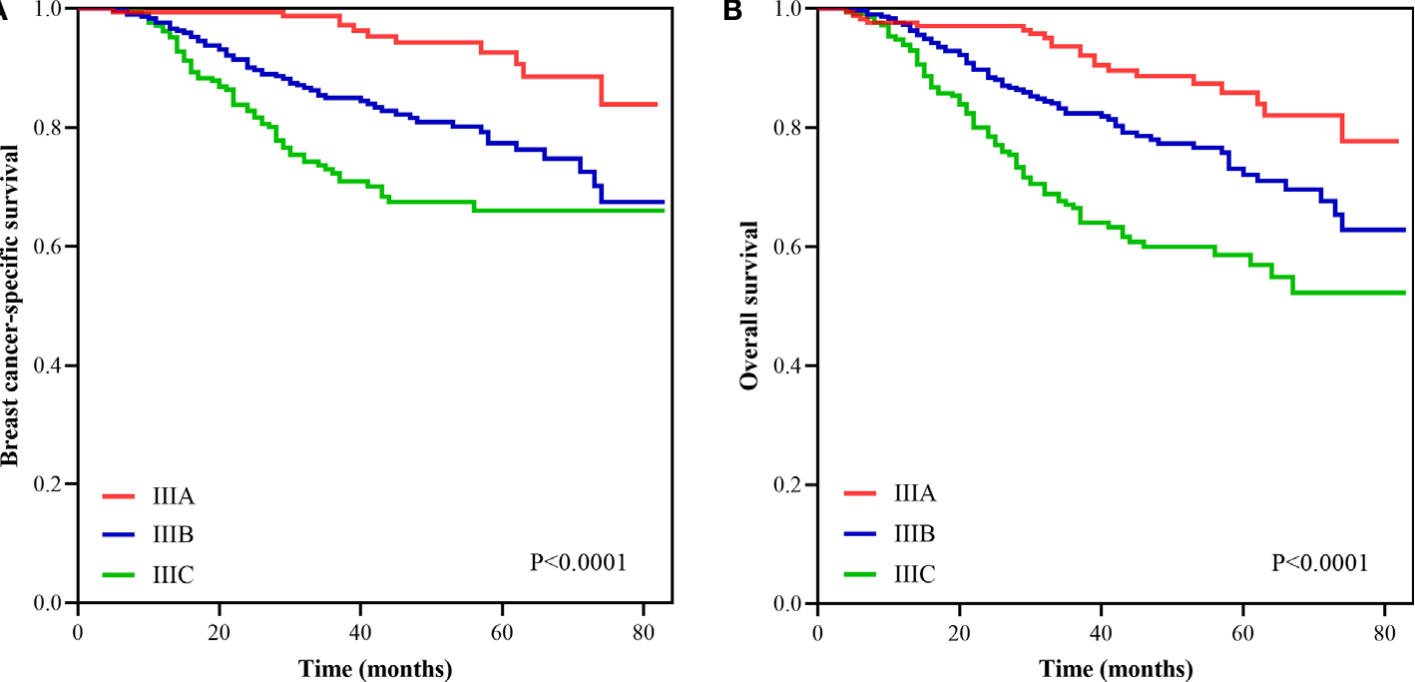
Survival curves according to different stages using the 8th edition of the American Joint Committee on Cancer pathological prognostic staging system (**A**, breast cancer-specific survival; **B**, overall survival).

**Figure 3:**
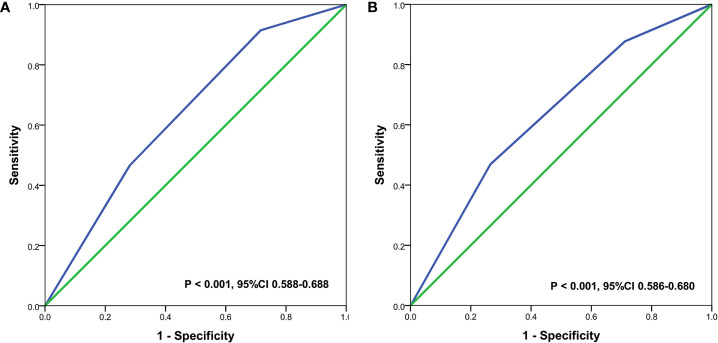
Receiver operating characteristics analyses for prediction the breast cancer-specific survival **(A)** and overall survival **(B)** using the 8th edition of the American Joint Committee on Cancer pathological prognostic staging system.

With a median follow-up of 41 months (interquartile range=30–59 months), the BCSS and OS rates at 5-years of the entire cohort were 77.8 and 71.4%, respectively (**Figure 1**). The new pathological prognostic staging system had better discriminatory value on prognostic prediction among IMN-metastasized breast cancer patients, with a 5-year breast cancer-specific survival (BCSS) of 92.7, 77.4, and 66.0% in stage IIIA, IIIB, and IIIC diseases, respectively (P<0.0001) (**Figure 2A**), and the 5-year overall survival (OS) rates was 85.9, 72.1, and 58.7%, respectively (P<0.0001) (**Figure 2B**). The ROC analysis showed that the pathological prognostic staging demonstrated moderate discriminative ability in predicting the BCSS (AUC 0.638, 95%CI 0.588–0.688, P<0.001) and OS (AUC 0.633, 95%CI 0.586–0.680, P<0.001) (**Figure 3**).

Two prognostic models were used to assess the independent prognostic factors associated with BCSS and OS. In the first model, including the biologic factors in the multivariate prognostic analysis, the results showed that infiltrating lobular carcinoma subtype, higher tumor grade (G3), HER2-negative, and ER-negative were the independent adverse prognostic factors related to inferior BCSS (**Table 3**). Moreover, older age (aged ≥50 years), infiltrating lobular carcinoma subtype, HER2-negative, and ER-negative were the independent adverse prognostic factors related to inferior OS (**Table 3**). In the second model, the 8th AJCC pathological prognostic staging was included in the multivariate prognostic analysis, and the results indicated that the pathological prognostic staging was the independent prognostic factors related to BCSS and OS, the 8th AJCC pathological prognostic stages showed worse BCSS and OS with gradually increased hazard ratios (**Table 4**).

**Table 3:** Multivariate analysis of prognostic factors using the Cox-regression model (including biologic factors).

Variables	BCSS		OS	
	HR (95% CI)	P	HR (95% CI)	P
Age (years)				
≥50 vs. <50	1.070(0.743–1.543)	0.715	1.465(1.062–2.019)	0.020
Race/ethnicity				
Non-Hispanic Black vs. Non-Hispanic White	1.223(0.790–1.895)	0.367	1.272(0.865–1.870)	0.222
Hispanic (All Races) vs. Non-Hispanic White	0.736(0.396–1.369)	0.333	1.015(0.615–1.676)	0.953
Other vs. Non-Hispanic White	0.784(0.401–1.532)	0.476	0.761(0.415–1.398)	0.379
Histology				
ILC vs. IDC	2.567(1.278–5.155)	0.008	2.339(1.275–4.292)	0.006
Other vs. IDC	0.791(0.421–1.484)	0.465	0.870(0.527–1.438)	0.587
Histologic grade				
G2 vs. G1	1.638(0.375–7.148)	0.512	0.724(0.309–1.697)	0.458
G3 vs. G1	4.932(1.169–20.804)	0.030	1.741(0.768–3.947)	0.184
T category				
T2 vs.T1	0.462(0.263–0.813)	0.007	0.698(0.430–1.133)	0.146
T3 vs.T1	1.235(0.727–2.099)	0.434	1.202(0.732–1.975)	0.466
T4 vs.T1	1.172(0.675–2.034)	0.573	1.368(0.834–2.246)	0.215
HER2 status				
Positive vs. Negative	0.588(0.385–0.899)	0.014	0.549(0.376–0.802)	0.002
ER status				
Positive vs. Negative	0.640(0.440–0.931)	0.020	0.568(0.408–0.793)	0.001
PR status				
Positive vs. Negative	0.779(0.474–1.281)	0.326	0.747(0.484–1.155)	0.190

ILC, infiltrating lobular carcinoma; IDC, infiltrating ductal carcinoma; BCS, breast-conserving surgery; BCSS, breast cancer-specific survival; ER, estrogen receptor; G1, well-differentiated; G2, moderately differentiated; G3, poorly/undifferentiated; HER2, human epidermal growth factor receptor-2; MAST, mastectomy; OS, overall survival; RT, radiotherapy; PR, progesterone receptor; T, tumor.

**Table 4:** Multivariate analysis of prognostic factors using the Cox-regression model (including 8th AJCC pathological prognostic staging).

Variables	BCSS		OS	
	HR (95% CI)	P	HR (95% CI)	P
Age (years)				
≥50 vs.<50	1.060 (0.742–1.516)	0.748	1.438(1.048–1.974)	0.024
Race/ethnicity				
Non-Hispanic Black vs. Non-Hispanic White	1.291 (0.837–1.990)	0.248	1.298(0.886–1.904)	0.181
Hispanic (All Races) vs. Non-Hispanic White	0.813 (0.440–1.504)	0.510	1.029(0.627–1.691)	0.909
Other vs. Non-Hispanic White	0.770 (0.396–1.495)	0.440	0.765(0.419–1.397)	0.383
Histology				
ILC vs. IDC	2.113 (1.089–4.102)	0.027	2.176(1.236–3.832)	0.007
Other vs. IDC	0.740 (0.396–1.382)	0.344	0.886(0.539–1.457)	0.634
AJCC pathological prognostic staging				
IIIB vs. IIIA	3.460 (1.802–6.644)	<0.001	2.209(1.346–3.625)	0.002
IIIC vs. IIIA	6.026(3.115–11.657)	<0.001	4.389(2.668–7.219)	<0.001

AJCC, American Joint Committee on Cancer; BCSS, breast cancer-specific survival; CI, confidence interval; HR, hazard ratio; IDC, infiltrating ductal carcinoma; ILC, infiltrating lobular carcinoma; OS, overall survival.

## Discussion

IMN-metastasized breast cancer is a unique subgroup with biological heterogeneity, whereas the influences of biological factors on staging migration and prognosis prediction were not fully explored for this population. Thus, to distinguish these patients and accurately predict prognosis, the current study analyzed the clinical significance of the AJCC 8th breast cancer staging system for IMN-metastasized patients. In our study, the new pathological prognostic staging system showed that 68.4% of patients were downstaged to stage IIIA or IIIB using the 8th AJCC criteria, and the 8th AJCC pathological prognostic stages showed worse BCSS and OS with gradually increased hazard ratios. Our results showed that the 8th AJCC staging system also hold true in BC patients with IMN metastasis.

In the era of precision medicine, oncologists believe that biological markers are as important as tumor burden. The 8th BC pathological prognostic staging system of AJCC integrated four biological factors (tumor grade, ER, PR, and HER2 status) and anatomical TNM factors. This new staging system has been verified by many researchers in various breast cancer subtypes (13–21). In our study, 68.4% of patients were downstaged to IIIA and IIIB diseases using the 8th AJCC criteria, which was similar to the results from Joo et al. (61%) (16). The 8th AJCC pathological prognostic staging enabled a distinct classification of prognosis based on disease stage subtype compared to that observed using the traditional anatomical staging.

To the best of our knowledge, our study was the second study to assess the role of the 8th AJCC staging system in IMN-metastasized breast cancer patients. A previous study from Korea only included 66 patients with IMN metastasis, and they found significant differences in locoregional recurrence rate, distant metastasis-free survival, progression-free survival, and OS after stratification by the new stages (16). However, the survival outcomes were comparable between stage IIIA and IIIB disease using the Cox-regression model (16). In our study, a large sample size of patients in this rare BC subgroup were included (n=678), and we found that worse survival outcomes with gradually increased hazard ratios in the 8th AJCC staging system. Our results showed that the 8th AJCC pathological prognostic staging provided accurate prognostic information for this population. In addition, the application of the new AJCC staging system will help update follow-up strategies, make treatment decisions, and make risk stratification when designing clinical trials in the future.

Currently, ER and PR are known as biomarkers for guiding endocrine therapy and can provide information on prognosis. Compared with ER-negative and PR-negative tumors, those patients with ER-positivity or PR-positivity tend to be associated with lower stage and have a better prognosis (23–26). In our study, patients with ER-positive disease were also associated with better survival outcomes in BC patients with IMN metastasis. Moreover, we found that all patients with double HoR-positive tumors were downstaged. According to the study from Wang et al. (15), including patients with IIIA–IIIC tumors according to the 7th AJCC anatomical staging criteria, 57.1% of the double HoR-positive tumors were downstaged, whereas only 0.1% of the single HoR-positive tumors were downstaged. A study also suggested that the double HoR-positive subgroup exhibited better survival outcomes than those with single HoR-positive tumors (27). Although our study found that PR-negative tumors did not have prognostic value for this population, the significant differences in the survival curves allowed us to assume that the nonsignificant* *difference of PR status on prognosis did not bias our risk estimates using the new staging.

HER2-positive status instead of HER2-negative status should be deemed as a better prognostic factor in the era of anti-HER2 therapy (28). In our study, 100% of HER2-positive patients were downstaged to IIIA or IIB regardless of HoR status, while 43.9% of HER2-negative patients had their stages unchanged after using the novel prognostic staging system of BC. However, of the HER2-negative subtype, TNBC is a unique subset. In the present study, 99.3% of the TNBC subtype patients had their stages unchanged, and only 1 TNBC patient was downstaged, which might lead to a surmise that the new BC prognostic staging system did not show superior abilities to distinguish the prognoses of TNBC patients in comparison with the traditional anatomical stages of BC. A study (n=31,941) from He et al. also challenged the validity of the AJCC 8th breast cancer staging system in stage I to IIIC TNBC patients (18). Therefore, the validity of the new AJCC breast cancer staging system in IMN-metastasized TNBC patients is in question, and further studies of large cohorts are needed.

Histologic grade was an important prognostic factor for BC patients, including patients with IMN metastasis (29–32). Our study also showed that the tumor grade was an independent prognostic factor in IMN-metastasized BC. Our study showed that 45.6% of patients with G3 disease have their stages unchanged, while all patients with G1 disease and 94.6% of patients with G2 disease were downstaged. Therefore, histologic grade plays a significant role in giving accurate staging and should be integrated into the novel pathological prognostic staging system.

Most patients determined in the AJCC 8th breast cancer staging system received multimodal therapy, including chemotherapy, hormone therapy, and anti-HER2 therapy in the era of pluralistic treatment. Therefore, the prognoses reflected by the new pathological prognostic staging system were the prognoses in the context of comprehensive and standardized treatment based on the unique tumor characteristics and biologic behaviors of each patient (12, 33). Although patients included in our study did not have a record on hormone therapy and anti-HER2 therapy, most patients in our study received chemotherapy, and our results proved that the new AJCC breast cancer staging system could accurately evaluate the prognoses of these N3b patients. So, we can assume that most patients in our study received corresponding adjuvant therapy. In addition, it should be noted that the premise of the AJCC 8th edition stages can accurately predict the prognosis is the routine utilization of anti-HER2 therapy. Therefore, the newly proposed AJCC staging system should be applied with caution in areas where anti-HER2 therapy cannot be routinely applied.

The primary strength of this study was that we investigated the incorporation of the biologic factors into staging for patients with internal mammary lymph nodes metastasis, which may help to further define this framework. However, several limitations should be acknowledged in this study. Firstly, our study was retrospective, and the inherent biases of a retrospective study should not be neglected. Secondly, there was no record on chemotherapy regimen, hormone therapy, anti-HER2 treatment, radiotherapy dose, and radiotherapy volume in the SEER database, which might impact the prognostic estimates. Thirdly, we limited our study to patients diagnosed between 2010 and 2014 because the information regarding HER2 status was initiated in 2010, which led to a relatively short follow-up period. Moreover, 28.5 and 8.3% of the patients did not receive postoperative radiotherapy and chemotherapy, respectively, which may influence the prognostic assessment of this patient subset. Finally, the patients’ information extracted from the SEER program should be validated with a larger cohort, especially from the Chinese population. Due to the rarity of this specific BC subgroup, a larger cohort study included patients’ data from multiple institutes should be performed to validate our findings.

## Conclusion

In conclusion, our study suggests that IMN-metastasized BC is an independent disease entity whose prognosis depends on biological characteristics. The AJCC 8th pathological prognostic staging system could provide more refined prognostic stratification for IMN-metastasized BC patients. Longer follow-up studies are needed to confirm the validity and accuracy of its prognostic prediction and to customize a treatment program in IMN-metastasized BC patients.

## Data Availability Statement

Publicly available datasets were analyzed in this study. This data can be found here: https://seer.cancer.gov/.

## Ethics Statement

Ethical review and approval was not required for the study on human participants in accordance with the local legislation and institutional requirements. Written informed consent for participation was not required for this study in accordance with the national legislation and the institutional requirements.

## Author Contributions

C-LL, H-YZ, JW, Y-XC, and S-GW are lead authors who participated in data collection, manuscript drafting, tables/figures creation, and manuscript revision. S-GW aided in data collection. C-LL, H-YZ, JW, JL, and LH are senior authors who aided in drafting the manuscript and manuscript revision. Y-XC and S-GW are tahe corresponding authors who initially developed the concept and drafted and revised the manuscript. All authors contributed to the article and approved the submitted version.

## Funding

This work was partly supported by the Commission Young and Middle-aged Talents Training Project of Fujian Health Commission (No. 2019-ZQNB-25) and the National Natural Science Foundation of Fujian Province (No. 2020J011240).

## Conflict of Interest

The authors declare that the research was conducted in the absence of any commercial or financial relationships that could be construed as a potential conflict of interest.
